# Novel feeding system to promote establishment of breastfeeds after preterm birth: a randomized controlled trial

**DOI:** 10.1038/jp.2015.184

**Published:** 2015-12-10

**Authors:** K Simmer, C Kok, K Nancarrow, A R Hepworth, D T Geddes

**Affiliations:** 1 Centre for Neonatal Research and Education, School of Pediatrics and Child Health, King Edward Memorial Hospital, Perth, WA, Australia; 2 School of Paediatrics and Child Health, Faculty of Medicine, Dentistry and Health Sciences, The University of Western Australia, Perth, WA, Australia; 3 School of Chemistry and Biochemistry, Faculty of Science, The University of Western Australia, Perth, WA, Australia

## Abstract

**Objective:**

We aimed to determine if a novel feeding system where milk only flowed when the preterm infant created a vacuum would influence time to full oral feeds, the length of stay (LOS) in hospital and breastfeeding at discharge.

**Study Design:**

This was a randomized controlled trial in the tertiary neonatal intensive care unit at King Edward Memorial Hospital, Perth, Australia. Eligibility criteria were: preterm infants of gestational age 25 to 34 weeks receiving >75% human milk by gastric tube. Infants were randomly assigned to being fed with a novel teat (NT) or conventional teat (CT). Intention to treat analysis was performed.

**Result:**

Time to full suck feeds was not different between groups. LOS was shorter (mean: 2.5 days; *P*=0.026) and less formula was fed at discharge in the NT group (*P*=0.036).

**Conclusion:**

Use of a NT that releases milk when the infant applies vacuum while establishing breastfeeding reduces duration of hospitalization of preterm infants.

## Introduction

The importance of human milk for preterm infants cannot be understated and include less gastrointestinal disease, improved neurodevelopment, higher metabolic protection, as well as both protection from infection and development of the neonatal immune system.^1, 2, 3, 4^ Further the economic benefits of human milk are dose dependent in both the term and preterm population.^5, 6^ Therefore, it is important to expedite full maternal milk production to not only improve infant health but increase the chances of successful breastfeeding thereby reaping the long-term benefits of human milk. However, preterm infants owing to their developmental immaturity and other co-morbidities often cannot feed at birth and have difficulties establishing breastfeeding.^7^ There is little available information on how best to establish breastfeeding in preterm infants.

Owing to the preterm infants sucking immaturity the majority of preterm infants are fed, at least initially via an oro- or nasogastric tube as it is imperative good growth is achieved as this is linked to improved cognitive outcomes. Breastfeeding is introduced as soon as the infant is stable enough,^8^ however, in the absence of the mother the mode of feeding becomes a clinical dilemma. In many units bottle feeding is practiced to expedite achievement of full oral feeds, which is often a requirement for discharge from hospital. Bottles are not recommended during the establishment of breastfeeding owing to increased flow rates and teat configurations increasing the possibility of ‘nipple confusion', where the infant refuses to feed from the breast preferring the bottle, despite little evidence of this scenario^9^ (BFHI^10^).^5^ One randomized controlled trial^11^ found that breastfeeding rates were higher 6 months post partum in a group that received intragastric tube supplementation and ongoing support from skilled lactation professionals compared with those that received a bottle. Alternative feeding methods include cup feeding, which is reportedly associated with higher breastfeeding rates, however, has serious impediments such as loss of substantial volumes of milk and requires skilled clinicians to deliver the feed.^12, 13^

One potential solution would be to design a system that more closely resembles the sucking dynamics of breastfeeding. Since vacuum has a major role in milk removal from the breast^14^ in term infants and increases efficacy of feeding in bottle-fed preterm infants,^15^ a teat allowing milk to flow only upon the application of vacuum may encourage more rapid maturation of feeding. Further, this would enable infant regulation of milk flow reducing negative consequences such as gagging, choking, bradycardia and oxygen desaturation episodes.^15^

The aim of our study was to test a novel feeding system designed to simulate a sucking mechanism comparable to breastfeeding in a randomized controlled trial. The teat was designed to release milk when vacuum was applied as well as to encourage a tongue motion similar to that of term breastfed infants. Primary outcome measures were time to first and full suck feeds, length of stay (LOS) and breastfeeding at discharge. A secondary outcome was breastfeeding rates at 3, 6 and 12 weeks post discharge from hospital.

## Methods

The study was conducted at King Edward Memorial Hospital (KEMH) and approved by the Ethics Committee of the Women's and Newborns' Health Service in Western Australia. KEMH is the only tertiary perinatal centre in Western Australia.

Informed written consent was obtained from the parents. This trial was a randomized controlled trial where participants were randomized to one of two parallel groups with balanced randomization (1:1) and was registered at the Australian New Zealand Clinical Trials Registry, ACTRN12614000875606, http://www.ANZCTR.org.au/ACTRN12614000875606.aspx.

Inclusion criteria were infants: gestational age (GA) 25 to 34 weeks; whose mothers intended to breastfeed; and who required 75% enteral feeds by intragastric tube with the remainder provided by parental nutrition. Exclusion criteria were: congenital anomalies, grade 4 intra-cerebral hemorrhage and periventricular leukomalacia and oral anomalies (for example, ankyloglossia, cleft palate). Primary outcomes were: time to first and full suck feeds, LOS and breastfeeding at discharge. Secondary outcomes were breastfeeding rates at 3, 6 and 12 weeks post hospital discharge ascertained by telephoning the mother at the prescribed time points post discharge of the infant home. Criteria for discharge from hospital to home were full suck feeds for a minimum of 48 h without weight loss, caffeine administration ceased, cardiorespiratory stability (no apnea or bradycardia) for at least 5 days.

The intervention group (novel teat (NT)) used a novel feeding system (Medela AG, Baar, Switzerland) that combined strategies known to improve oral feeding skills: development of vacuum^15^ and self-paced feeding.^16^ A shut-off valve was incorporated to ensure milk flowed only when the infant created a vacuum and venting prevented collapse of the teat. There were two different threshold levels for the valve of −10±5 mm Hg and −30±15 mm Hg (−30 mm Hg is similar to the commercially available Calma teat, Medela AG, Baar, Switzerland). The control group (conventional teat (CT)) used a CT that allowed milk flow with gravity and compression of the teat (Grow, Growbaby, Icon Health, Victoria, Australia or Peristaltic narrow Neck Slow Flow, Pigeon, Seoul, South Korea).

Infants were randomized when they reached full enteral feeds with at least 25% of feeds being delivered as suck feeds (by KC, KN). Sealed opaque coded envelopes containing the computer generated treatment allocation were sequentially numbered for randomization (by biostatistician ARH). Recruitment and separate randomization into two subgroups of infants 25 to 29^+6^ weeks and 30 to 33^+6^ weeks GA was performed. Twins were considered as individuals but randomized to the same arm to ensure compliance. As per ethics approval we endeavored to enroll all eligible infants to achieve statistical power to detect a decrease in LOS in hospital of 3 days. In all, 30 infants were initially enrolled (15 in each group) as an internal pilot to determine sample size. Following the pilot study a sample size of 30 in each group, gave sufficient power to show a difference in LOS of 3 days.

Bottles were offered only if a bottlefeed was scheduled and duration of the feed was limited to 30 min. Infants were not fed ad libitum. Non-nutritive sucking is encouraged up to 33 weeks before suck feeds after which increasingly suck feeds replaced non-nutritive sucking. Owing to the nature of the feeding device, blinding of staff and parents to allocated treatment was not possible. Safety was assessed with all infants' vital signs recorded as per neonatal intensive care unit guidelines. Records were reviewed after 25, 50, 75 and 100% of enrollment. Infants were transferred when appropriate from KEMH to secondary hospitals.

Analysis of the data was carried out by a biostatistician (ARH) who was not involved in any of the data collection and who was blinded to treatment allocation. Statistical analysis was performed using R 2.9.0 for Mac OS X^17^ with packages nlme^18^ and lattice.^19^ A *P*-value of <0.05 was considered significant. Data are presented as mean±s.d. unless otherwise specified. Analysis was done on an intention-to-treat basis.

### Statistical analysis

Modeling of continuous variables used linear mixed effects models in order to account for the related nature of the data, given that twins from the same pregnancy were assigned to the same treatment group, with differences in baseline values for a family group as the random effect. In multivariate models, treatment group was included even when not significant.

Groups were compared on demographic and introduction of suck feed variables using univariate linear mixed effects models or Fisher's exact test, for continuous and categorical data, respectively, to determine whether differences had arisen despite randomized allocation. Predictor in the mixed effects models was treatment group.

Multivariate linear mixed effects models were determined for achievement of full suck feeds and discharge variables. Variables for the initial model were determined by identifying all predictors that were significant after accounting for treatment group effects. The final model was selected by sequentially omitting non-significant variables until all remaining variables had marginal *P*-values <0.05, and then testing for significance of each of the omitted variables when added to this model. Where additional significant variables were found, this was then repeated.

Considered covariates for the achievement of suck feed variables were the corrected gestational age (CGA) at introduction of suck feeds, the CGA at introduction of teat feed, infant multiplicity, BGA, birth weight, whether the mother was a primigravida, whether the mother had previously breastfed, whether the mother developed mastitis, use of oxygen, and duration of ventilation and continuous positive airway pressure.

Considered covariates for the discharge variables were CGA at introduction of first suck feeds, the CGA at introduction of teat feeds, the number of days from first to full sucks, the CGA at full suck feeds, infant multiplicity, BGA, birth weight, use of oxygen, duration of ventilation and continuous positive airway pressure, and whether the infant was discharged home or to a peripheral hospital.

Owing to the uneven spread of the data, the three respiratory support variables were converted to categorical variables. Oxygen use was classified as ‘yes' or ‘no' continuous positive airway pressure duration as ‘⩽1 week' and ‘>1 week' and ventilation as ‘never', ‘⩽48 h' and ‘>48 h'.

Breastfeeding at discharge was grouped into a number of dichotomous variables, and frequency in the two groups compared using Fisher's exact test. Where significance was *P*<0.10, relative risk (RR) (odds ratios, 95% confidence intervals (CI)) were determined using logistic regression models. *P*-values reported are from Fisher's exact test, not the logistic regression, as they are the more conservative values. Feed type at each follow-up point was a four-category factor, and was compared between groups using Fisher's exact test.

Analyses were repeated for two subsets of the data, being those who were correctly enrolled in the study and received either intervention or control teats in the tertiary center (partial protocol/PP, *n*=78), and for those who received the assigned intervention until discharge home (complete protocol/CP, *n*=67). Results from these analyses are presented in the Supplementary tables. (PP, *n*=78; Supplementary Tables S1 and S2).

## Results

**Figure 1:**
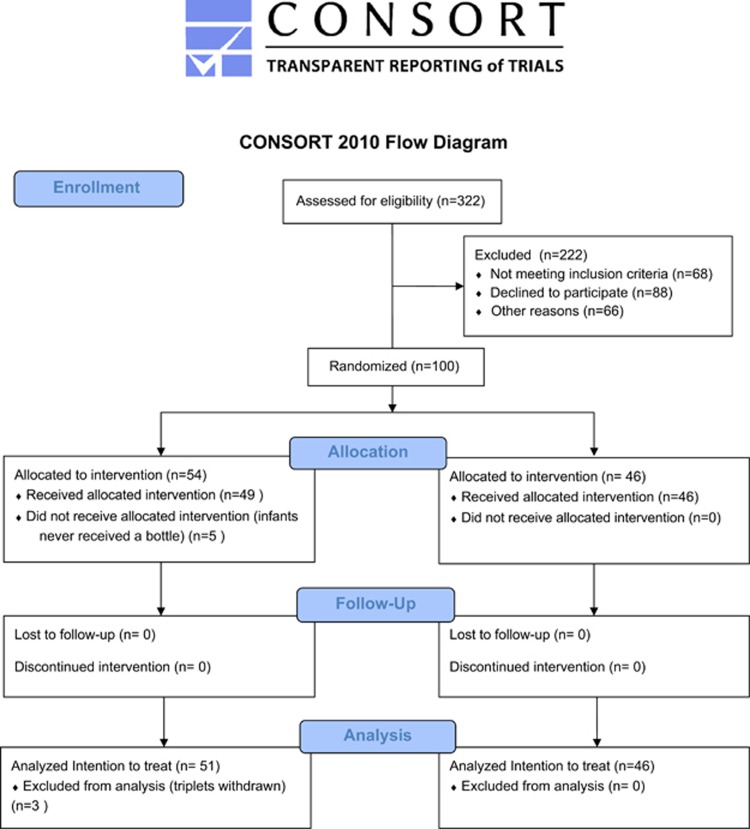
CONSORT 2010 flow diagram.

The study period was 1 August 2011 to 30 June 2012. In all, 100 infants were enrolled, 3 withdrew leaving 97 (NT: *n*=51; CT: *n*=46) in the intention-to-treat analysis. Nineteen were excluded: 10 were never bottle-fed; 4 were too ill; 4 ceased breastfeeding and 1 did not meet GA criterion. In all, 78 infants remained (PP); 29 were discharged home from KEMH and 49 transferred to a secondary hospital. Of the 49 transferred, 11 in the NT were excluded for receiving the control teat leaving 67 infants (CP; Figure 1).

Infant characteristics and demographics were not different between groups (Table 1). The CP NT had more twins (47 vs 23%, *P*=0.044) and more mastitis (22 vs 0%, *P*=0.009, Supplementary Table S1) and the PP had more mastitis (16 vs 0%, *P*=0.017).

There was no difference in CGA for introduction of suck feeds or teat feeds in each group (Table 2). Infants received the low vacuum threshold teat with six offered the higher threshold teat in the days before discharge.

Age at full suck feeds (*P*=0.22), the time from first suck to full suck feeds (*P*=0.23) and time from first teat to full suck feeds (*P*=0.52; Table 2) did not differ between the groups. Infants <30 weeks GA established full suck feeds later than those ⩾30 weeks GA with no difference between subgroups (NT: 38^1^±2^1^, *n*=20; CT: 39^1^±2^1^, *n*=19, CGA <30 weeks *P*=0.234; NT: 36^3^±1, *n*=31; CT: 36^2^±1^6^, *n*=27 CGA ⩾30 weeks, *P*=0.989).

By univariate analysis LOS (NT: 7.6±4.1; CT: 8.3±4.7 weeks, *P*=0.62) and CGA (NT: 37^5^±1^6^, CT: 38^3^±2^3^ weeks, *P*=0.24) at discharge home were similar between groups. The NT had a significantly lower weight at discharge home (*P*=0.002, Table 3)

Accounting for potential confounding variables, the NT had shorter LOS overall being discharged home on average 2.5 days earlier (*P*=0.032), at a younger CGA (*P*=0.024) and lighter weight (*P*=0.001; Table 3).

At discharge from the KEMH, breastfeeding (NT: 96% CG: 78%, *P*=0.012, RR 6.8, 95% CI 1.7, 46.0) and breast milk feeding rates (NT: 98% CG: 83%, *P*=0.012, RR 10.5, 95% CI 1.8, 200) were higher in the NT, and formula use lower (NT: 14% CG: 33%, *P*=0.031, RR 0.33, 95% CI 0.11, 0.87). At discharge home, there was no difference in breastfeeding rates (NT: 90% CG: 76%, *P*=0.10) and the difference in formula feeding rates remained (NT: 16% CT: 35%, *P*=0.036, RR 0.35, 95% CI 0.13, 0.90; Table 4). Rates of breastfeeding after discharge from KEMH or the secondary hospital was similar in the two groups (Table 5). At 3 weeks post discharge reports of any breastfeeding was 84% for the NT and 74% for CT. The breastfeeding rate fell to 78% and 67%, (*P*=0.26) at 6 weeks and 55 and 46%, (=0.40) at 12 weeks post discharge for NT and CT respectively.

No concerns about safety of the novel teat were reported by either the parents or the nursing staff.

## Discussion

Infants who fed with the novel teat had a shorter LOS and were breastfeeding more at discharge, although time to full suck feeds were not different to the CT. In keeping with the shorter LOS, infants fed with the novel teat were discharged younger and lighter compared with those fed with a CT. All eligible infants were enrolled, and this is reflected in that ~60% of infants required respiratory support. Hence, these results have applicability to the wider neonatal intensive care unit population.

One of the criteria for discharge of preterm infants home from hospital is achievement of full suck feeds. Despite this milestone not being met any earlier, infants fed with the novel teat in this study were discharged home 2.5 days earlier. Earlier discharge home has multiple positive effects such as psychosocial benefits to the family^20^ and reduced risk to the infant,^21^ therefore every effort is made to expedite infant discharge. Further any reduction in LOS confers significant economic benefits to the health system.^22^

Given the earlier discharge of the infants fed with the novel teat, it follows that they were lighter than the control group by 185 to 245 g. This difference was greater than that of the birth weights of the infants in each group (NT: 1310±442, CT: 1430±507 g, *P*=0.261; Table 1). One limitation of the study is that milk intake from the breastfeeds was not measured in either group, therefore it is remotely possible that this group received a lower total volume of milk overall, contributing in part to their lighter discharge weight.^23^ More energy might have been required to suck from the novel teat than the CT, which allows infants to express milk from the teat without applying vacuum.^24^ However, a recent study suggests resting energy expenditure is similar for preterm breast and bottle feeding infants.^25^ Nevertheless, these infants met the discharge criteria of full suck feeds including adequate growth, and satisfactory breastfeeding indicating that the novel teat more likely accelerated the development of sucking via entraining.^26^ Further investigation is required to clarify if this is the mechanism associated with early discharge.

Lau *et al.*^15^ examined feeding development of bottle-fed preterm infants by measuring sucking vacuum and improved feeding performance was associated with maturation of sucking skills. Sucking pressures decreased (increased in strength) with advancing age from −10 mm Hg to −100 mm Hg, a vacuum that is equivalent to the term breastfed infant.^14, 27, 28^ The vacuums applied by our infants were sufficient to open the shut-off valve (–10 or −30 mm Hg) and is equivalent to the baseline vacuum applied by the breastfeeding infant.^29^ Staff rarely chose to feed with the high threshold teat, which may reflect reduced intra-oral vacuums^15^ and/or infant state at feeding.^30^ Future trials will determine optimum timing of the introduction of the high threshold teat.

The first suck feed (normally a breastfeed) was introduced relatively late at 333 weeks CGA in our study, and first teat feed at 343 weeks CGA, despite a policy to introduce oral feeds when the infant exhibits cues of developmental readiness.^30, 31^ Full suck feeds were achieved at 37 to 38 weeks CGA. Had the infants begun suck feeds earlier they may have subsequently achieved full feeds earlier. In an randomized controlled trial^32^ of infants born <30 weeks GA (*n*=29), those that began suck feeds at 31 weeks CGA achieved full feeds 10 days earlier than those who began at 34 weeks CGA, with no difference in weight gains. Thus earlier introduction of oral feeds may have enhanced motor skill development, contributing to more rapid maturation of feeding. Similarly earlier introduction of breastfeeds^6^ and oral stimulation also shortens the time to full oral feeding.^26, 33^ Further studies examining the effect of earlier introduction of the novel teat on establishment of full suck and breastfeeds are warranted.

One of the unique features of the novel teat is that it allows infants to independently control the flow of milk, whereas CTs flow under the influence of gravity and therefore could potentially improve cardiorespiratory stability. As with the breast, infants fed with the novel teat were able to stop milk flow by raising their tongue^34, 35^ and ceasing sucking (milk can not flow under gravity). Greater control of milk intake from the novel teat by the infant may lead to more stable oxygenation and temperature similar to breastfed infants.^36^ Better cardiorespiratory responses in the NT may therefore have led to earlier discharge, however these were not reliably or systematically recorded and should be investigated in future studies.

We speculated the novel teat would facilitate breastfeeding and importantly, at discharge rates were significantly higher from the tertiary center (96% vs 78%) with more breast milk fed (Table 5). At discharge home breastfeeding rates remained higher, although not significantly so, which may be due to lower compliance with the intervention and/or differing breastfeeding policies of secondary hospitals.

Although the majority (90%) of preterm infants were breast/breast milk feeding at discharge home 35% infants were fully breastfeeding with 50% breastfeeding 12 weeks post discharge irrespective of group. Although long-term breastfeeding rates were disappointing, several factors are likely to have influenced this, such as lower compliance at the secondary hospital, maternal intention to breastfeed and the inability to achieve or sustain a full milk production.^37^ Most importantly, cessation of the trial at discharge home means the continued use of the novel teat is unknown and given prior evidence of a training effect for maturation of feeding^26, 33, 38^ it is possible continuation may have been beneficial and warrants further investigation.

Ours was a pragmatic trial and the associated complexities of preterm infants were considered in the multivariate analysis. The limitations were the impossibility of blinding staff and mothers from the intervention and that back transfers from KEMH to secondary hospitals could have reduced compliance.

## Conclusion

This study has shown that the use of a novel feeding system that releases milk when the infant applies vacuum in preterm infants reduces duration of hospitalization and increases breastfeeding at discharge. Although the system does not reduce time to achievement of first and full oral feeds and post discharge breastfeeding rates, these results suggest a training effect of the novel teat on feeding that should be further investigated.

## Figures and Tables

**Table 1:** Comparison of demographic factors between groups

	*Novel teat (*n=*51)*	*Conventional teat (*n=*46)*	P-*value*
*Maternal factors*			
Maternal age (y)	30.6±6.1	28.9±6.8	0.514
Gravidity			
Primigravida	25 (49)	17 (37)	0.305
Multigravida	26 (51)	29 (63)	
Maternal marital status			
Married	25 (49)	26 (56)	0.300
*de facto*	17 (33)	9 (20)	
Single	9 (18)	11 (24)	
Previous breastfeeding			
Yes	21 (41)	21 (46)	0.686
No	30 (59)	25 (54)	
Post-partum infections			
None	40 (78)	39 (85)	0.068
Mastitis^a^	9 (18)	2 (4)	
Other	2 (4)	5 (11)	

*Birth and infant factors*			
Multiplicity			0.137
Singletons	30 (59)	34 (74)	
Twins	21 (41)	12 (26)	
Delivery mode			0.198
Spontaneous vaginal delivery	14 (27)	19 (41)	
C/Section	37 (73)	27 (59)	
Birth weight (g)	1310±422	1430±507	0.261
Birth gestational age (wks)	30.1±2.7	30.1±2.6	0.847
Respiratory support-ventilation (h) none	26 (51)	21 (46)	0.337
⩽48 h	17 (33)	12 (26)	
>48 h	8 (16)	13 (28)	
Continuous positive airway pressure use			0.683
⩽ 1 week	30 (59)	29 (63)	
>1 week	21 (41)	17 (37)	
Oxygen use			
Yes	32 (63)	29 (63)	1.00
No	19(37)	17(37)	
Hours (median (min, max))	2(0, 2841)	8.5 (0, 2253)	0.57

*Chronic lung disease*			
Yes	8 (16)	9 (20)	0.79
No	43 (84)	37 (80)	
Caffeine R_x_			1.00
Yes	42 (82)	38 (83)	
No	9 (18)	8 (17)	
Late onset sepsis^b^			0.38
Yes	5 (10)	8 (17)	
No	46 (90)	38 (83)	

Abbreviation: SVD, spontaneous vaginal delivery. Data is presented as percentage for categorical variables, and mean±s.d. for continuous variables.

a This includes one individual where both mastitis and another post-partum infection occurred.

b Two infants in the conventional teat group had multiple episodes of sepsis; all other infants had a single episode.

**Table 2:** Comparisons of introduction of suck feeds and transition to full suck feeds between the two groups

	*Novel teat (*n=*51)*	*Control (*n=*46)*	*Univariate* P-*value*	*Treatment effect (days) estimate (95% CI)*	*Multivariate* P-*value*^a^
*Introduction of suck feeds*					
First suck feed					
CGA (wks) (a)	33.3±0.9	33.7±1.7	0.228	—	—
Days post partum	22±19	25.4±23.3	0.712	—	—
First teat feed					
CGA (b)^b^	34.5±1.0	34.7±1.4	0.531	—	—
Days post partum	31±22	33±23	0.925	—	—
Days between first suck and first teat feeds (b−a)^b^	8±7	8±7	0.477	—	—

*Achievement of full suck feeds*					
CGA (wks)	37.2±1.7	37.4±2.4	0.190	−2.8 (−7.3, 1.7)	0.217
Days					
Post partum	48±27	52±31	0.394	−2.7 (−7.2, 1.8)	0.240
From first suck feed	26±12	26±14	0.902	−2.7 (−7.2, 1.8)	0.234
From first teat feed	18±11	19±12	0.520	−2.8 (−7.3, 1.7)	0.218

Abbreviations: CI, confidence interval; CGA, corrected gestational age; wks, weeks.

Data are presented as mean±s.d., with *P*-values for univariate and multivariate models. Treatment effect is the average between groups difference after accounting for significant covariates, and are presented as novel teat relative to the control teat, such that negative values indicate that the occurrence in group A is earlier/younger (smaller values).

a Multivariate analysis was done for achievement of full suck feed variables, but not for introduction of suck feed variables.

b *n*=93, as CGA at first breastfeed was not recorded for four infants where breastfeeding had ceased before teat feeding was introduced.

**Table 3:** Comparisons of length of stay measures between the two groups

	*Novel teat (*n=*51)*	*Control (*n=*46)*	*Univariate* P-*value*	*Treatment effect (days, g) estimate (95% CI)*	*Multivariate* P-*value*
*Length of stay*					
CGA at discharge home (wks)	37.7±1.9	38.4±2.5	0.244	−2.5 (−4.7, −0.3)	0.024
Length of stay (birth to discharge) (wks)	7.6±4.1	8.3±4.7	0.622	−2.5 (−4.6, −0.2)	0.032
First suck to discharge home (days)	31±13	33±16	0.607	−2.2 (−4.4, −0.1)	0.044
First teat to discharge home (days)	23±11	26±14	0.288	−2.6 (−4.8, −0.5)	0.018
Full sucks to discharge home (days)^a^	5±4	7±4	0.248	−2.5 (−4.7, −0.3)	0.024
Discharge weight (g)	2392±383	2698±447	0.002	−186 (−317, −56)	0.006

Abbreviations: CI, confidence interval; CGA, corrected gestational age; wks, weeks.

Data are presented as mean±s.d., with *P*-values for univariate and multivariate models. Treatment effect is the average between groups difference after accounting for significant covariates, and are presented as novel teat relative to the control teat, such that negative values indicate that the occurrence in the novel teat is earlier/younger (smaller values).

a This model includes both CGA at achievement of full suck feeds and number of days to achieve full suck feeds, as both are significant without the other in the model, and lose significance together.

**Table 4:** Comparisons of feed type at discharge between the two groups

*Discharge home*	*Novel teat (*n=*51)*	*Control (*n=*46)*	P-*value*	*RR (95% CI)*
Fully breastfed	19 (37%)	16 (35%)	0.835	—
Any breastfeeding	46 (90%)	35 (76%)	0.100	—
Any breast milk feeds	46 (90%)	37 (80%)	0.248	—
Any tube feeding	0 (0%)	1 (2%)	—	—
Any formula	8 (16%)	16 (35%)	0.036	0.35 (0.13, 0.90)

Data is presented as count (percentage). RR plus 95% CI are presented only where there is a significant difference between groups.

**Table 5:** Comparisons of feed type at follow-up between the two groups

	*Novel teat (*n=*51)*	*Control (*n=*46)*	*Univariate* P-*value*
*Week 3*			0.122
Fully breastfed	8 (16%)	11 (24%)	
Breastfeeding plus EBM	29 (57%)	15 (33%)	
Breastfeeding plus formula	6 (12%)	8 (17%)	
Formula only	8 (16%)	12 (26%)	
*Week 6*			0.291
Fully breastfed	6 (12%)	9 (20%)	
Breastfeeding plus EBM	18 (35%)	10 (22%)	
Breastfeeding plus formula	16 (31%)	12 (26%)	
Formula only	11 (22%)	15 (33%)	
*Week 12*			0.423
Fully breastfed	7 (14%)	5 (11%)	
Breastfeeding plus EBM	6 (12%)	5 (11%)	
Breastfeeding plus formula	15 (29%)	11 (24%)	
Formula only	23 (45%)	25 (54%)	

Abbreviation: EBM, expressed breastmilk. Data is presented as count (percentage), *P*-values are overall comparison between the counts for the four categories.
