# Supplementary material for: Novel feeding system to promote establishment of breastfeeds after preterm birth: a randomized controlled trial
Source: J Perinatol. 2015 Dec 10;36(3):210–5. doi: 10.1038/jp.2015.184 (PMC4770056; doi:10.1038/jp.2015.184)
Supplement: Supplementary Table 1 [file jp2015184x1.docx]

**Supplementary Table 1: Univariate comparisons of demographic factors across the three data groupings, by treatment group. Data is presented as count (percentage within treatment group) for categorical variables, and mean ± SD for continuous variables. Note that Control group values are identical between the complete and partial protocols, as only the novel teat group varied on the inclusion criteria for this separation of the data.**

|  | Intention to treat  (n=97; 51 Novel teat, 46 Control) | | | | Partial Protocol  (n=78; 43 Novel teat, 35 Control) | | | Complete Protocol  (n=67; 32 Novel teat, 35 Control) | | | | |  |
| --- | --- | --- | --- | --- | --- | --- | --- | --- | --- | --- | --- | --- | --- |
|  | Novel teat | Control | p-value | | Novel teat | Control | p-value | Novel teat | | Control | | p-value | |
| **Maternal Factors** |  |  |  | |  |  |  |  | |  | |  | |
| Maternal age (y) | 30.6 ± 6.1 | 28.9 ± 6.8 | 0.514 | | 31.3 ± 5.9 | 29.5 ± 6.8 | 0.405 | 31.1 ±5.6 | | 29.5 ± 6.8 | | 0.652 | |
| Gravidity  Primigravida  Multigravida | 25 (49)  26 (51) | 17 (37)  29 (63) | 0.305 | | 20 (47)  23 (53) | 13 (37)  22 (63) | 0.492 | 12 (38)  20 (62) | | 13 (37)  22 (62) | | 1.00 | |
| Maternal marital status  married  de facto  single | 25 (49)  17 (33)  9 (18) | 26 (56)  9 (20)  11 (24) | 0.300 | | 21 (49)  16 (37)  6 (14) | 19 (54)  9 (26)  7 (20) | 0.532 | 16 (50)  13 (41)  3 ( 9) | | 19 (54)  9 (26)  7 (20) | | 0.313 | |
| Previous breastfeeding  Yes  No | 21 (41)  30 (59) | 21 (46)  25 (54) | 0.686 | | 19 (44)  24 (56) | 17 (49)  18 (51) | 0.820 | 16 (50)  16 (50) | | 17 (49)  18 (51) | | 1.000 | |
| Post-partum infections  None  Mastitis  Other | 40 (78)  9 (18)  2 ( 4) | 39 (85)  2 ( 4)  5 (11) | 0.068 | | 34 (79)  7 (16)  2 ( 5) | 31 (89)  0 ( 0)  4 (11) | 0.017 | 23 (72)  7 (22)  2 ( 6) | | | 31 (89)  0 ( 0)  4 (11) | 0.009 | |
| **Birth and Infant Factors** |  |  |  | |  |  |  |  | |  | |  | |
| Multiplicity  singletons  twins | 30 (59)  21 (41) | 34 (74)  12 (26) | 0.137 | | 24 (56)  19 (44) | 27 (77)  8 (23) | 0.059 | 17 (53)  15 (47) | | 27 (77)  8 (23) | | 0.044 | |
| Delivery mode  SVD  C/Section | 14 (27)  37 (73) | 19 (41)  27 (59) | 0.198 | | 12 (28)  31 (72) | 13 (37)  22 (63) | 0.467 | 11 (34)  21 (66) | | 13 (37)  22 (63) | | 1.00 | |
| Birthweight (g) | 1310 ± 422 | 1430 ± 507 | 0.261 | | 1308 ± 436 | 1420 ± 487 | 0.357 | 1245 ± 433 | | 1420 ± 487 | | 0.197 | |
| Respiratory support |  |  |  |  | |  |  | |  | |  |  | |
| Ventilation (h)  none  < 48 hours  longer | 26 (51)  17 (33)  8 (16) | 21 (46)  12 (26)  13 (28) | 0.337 | 22 (51)  14 (33)  7 (16) | | 19 (54)  8 (23)  8 (23) | 0.594 | | 16 (50)  9 (28)  7 (22) | | 19 (54)  8 (23)  8 (23) | 0.947 | |
| CPAP use  up to 1 week  longer | 30 (59)  21 (41) | 29 (63)  17 (37) | 0.683 | 25 (58)  18 (42) | | 23 (66)  12 (34) | 0.640 | | 17 (53)  15 (47) | | 23 (66)  12 (34) | 0.328 | |
| Birth gestational age (wks) | 30.1 ± 2.7 | 30.1 ± 2.6 | 0.847 | | 30.1 ± 2.7 | 30.2 ± 2.4 | 0.655 | 29.6 ± 2.8 | | 30.2 ± 2.4 | | 0.286 | |
