# Supplementary material for: Novel feeding system to promote establishment of breastfeeds after preterm birth: a randomized controlled trial
Source: J Perinatol. 2015 Dec 10;36(3):210–5. doi: 10.1038/jp.2015.184 (PMC4770056; doi:10.1038/jp.2015.184)
Supplement: Supplementary Table 2 [file jp2015184x2.docx]

**Supplementary Table 2: Univariate comparisons of timing of introduction of suck feeds across the three data groupings, by treatment group. Data is presented as mean ± SD for continuous variables. Note that Control group values are identical between the complete and partial protocols, as only the novel teat group varied on the inclusion criteria for this separation of the data**

|  | Intention to treat  (n=97; 51 Novel teat, 46 Control) | | | Partial Protocol  (n=78; 43 Novel teat, 35 Control) | | | Complete Protocol  (n=67; 32 Novel teat, 35 Control) | | |
| --- | --- | --- | --- | --- | --- | --- | --- | --- | --- |
|  | Novel teat | Control | p-value | Novel teat | Control | p-value | Novel teat | Control | p-value |
|  |  |  |  |  |  |  |  |  |  |
| First Suck (A)  CGA (wks) | 33.3 ± 0.9 | 33.7 ± 1.7 | 0.228 | 33.3 ± 0.9 | 33.4 ± 1.2 | 0.673 | 33.3 ± 0.9 | 33.4 ± 1.2 | 0.751 |
| Days PP | 22.4 ± 19.4 | 25.4 ± 23.3 | 0.712 | 22.3 ± 19.7 | 22.1 ± 18.6 | 0.757 | 25.6 ± 20.6 | 22.1 ± 18.6 | 0.362 |
| First Teat (B)* |  |  |  |  |  |  |  |  |  |
| CGA (wks) | 34.5 ± 1.0 | 34.7 ± 1.4 | 0.531 | 34.4 ± 1.0 | 34.6 ± 1.2 | 0.754 | 34.3 ± 1.1 | 34.6 ± 1.2 | 0.709 |
| Days PP | 30.5 ± 21.7 | 32.6 ± 23.2 | 0.925 | 30.1 ± 22.2 | 30.3 ± 20.8 | 0.817 | 32.9 ± 23.5 | 30.3 ± 20.8 | 0.480 |
| A-B * |  |  |  |  |  |  |  |  |  |
| Days | 8.2 ± 7.0 | 7.5 ± 6.9 | 0.477 | 7.7 ± 6.9 | 8.1 ± 6.3 | 0.802 | 7.2 ± 6.6 | 8.1 ± 6.3 | 0.576 |
|  |  |  |  |  |  |  |  |  |  |

* *Intention To Treat group has n=93, rather than n=97, as four infants were never given a teat feed in the tertiary centre.*
