# Supplementary material for: Novel feeding system to promote establishment of breastfeeds after preterm birth: a randomized controlled trial
Source: J Perinatol. 2015 Dec 10;36(3):210–5. doi: 10.1038/jp.2015.184 (PMC4770056; doi:10.1038/jp.2015.184)
Supplement: Supplementary Table 3 [file jp2015184x3.docx]

**Supplementary Table 3 – Significance (p-values) of selected predictors on the timing of outcomes of interest relating to discharge home. Note that not all predictors were considered to be predictive for all outcomes of interest. Differences between treatment groups are novel teat relative to the control teat, such that negative values indicate that the occurrence in the control group is earlier/younger [smaller values]. Bold text indicates significant variables retained in the explanatory model, and p-values for these variables comes from that model. P-values for other variables are for models containing the explanatory model variables plus that one variable. ITT indicates intention to treat analysis (all recruited infants), PP indicates partial protocol (protocol was followed until discharge from the tertiary centre), CP indicates complete protocol (protocol was followed until discharge home).**

|  | Treatment effect  (days) | Group | CGA 1^st^ suck | CGA first teat | Number of days to full sucks | CGA at full sucks | Multiplicity | BGA | Birthweight* | Ventilation  [never; <48 h, ≥48h] | Oxygen  [never, ever] | CPAP  [<1 week;  > 1 week] | Discharged home? |
| --- | --- | --- | --- | --- | --- | --- | --- | --- | --- | --- | --- | --- | --- |
| CGA at discharge home  *ITT*  *PP*  *CP* | -2.5  -3.0  -3.5 | **0.024**  **0.007**  **0.005** | **<0.001**  **<0.001**  **<0.001** | 0.367  0.847  0.545 | **<0.001**  **<0.001**  **<0.001** | -  -  - | 0.544  0.547  0.607 | 0.618  0.768  0.675 | **<0.001**  **0.028**  **0.028** | 0.486  0.548  0.284 | 0.473  0.654  0.546 | 0.205  0.339  0.226 | 0.845  0.587  0.779 |
| Length of hospital stay  *ITT*  *PP*  *CP* | -2.5  -3.1  -3.6 | **0.032**  **0.007**  **0.004** | **<0.001**  **<0.001**  **<0.001** | 0.385  0.812  0.488 | **<0.001**  **<0.001**  **<0.001** | -  -  - | 0.604  0.490  0.526 | **<0.001**  **<0.001**  **<0.001** | **0.006**  ***0.061***  ***0.088*** | 0.546  0.494  0.294 | 0.530  0.563  0.447 | 0.253  0.222  0.129 | 0.665  0.626  0.836 |
| Time from first suck to discharge  *ITT*  *PP*  *CP* | -2.2  -3.0  -3.3 | **0.044**  **0.008**  **0.007** | 0.242  0.644  0.339 | 0.782  0.856  0.721 | **<0.001**  **<0.001**  **<0.001** | 0.241  0.640  0.335 | 0.576  0.543  0.627 | 0.552  0.840  0.872 | **<0.001**  **0.021**  **0.029** | 0.485  0.642  0.371 | 0.521  0.670  0.587 | 0.247  0.338  0.265 | 0.642  0.548  0.678 |
| First teat to discharge  *ITT*  *PP*  *CP* | -2.6  -3.0  -3.5 | **0.018**  **0.007**  **0.006** | 0.778  0.733  0.313 | **<0.001**  **<0.001**  **<0.001** | 0.782  0.727  0.308 | **<0.001**  **<0.001**  **<0.001** | 0.572  0.526  0.548 | 0.706  0.862  0.909 | **<0.001**  **0.027**  **0.028** | 0.477  0.578  0.391 | 0.582  0.617  0.472 | 0.266  0.301  0.233 | 0.943  0.573  0.751 |
| Achievement of full sucks to discharge **  *ITT (A)*  *ITT (B)*  *PP*  *CP* | -2.6  -2.2  -3.0  -3.3 | **0.018**  **0.044**  **0.007**  **0.006** | 0.275  0.241  0.716  0.343 | 0.175  0.783  0.860  0.708 | -  **0.022**  0.940  0.819 | **0.018**  -  0.700  0.560 | 0.599  0.574  0.551  0.601 | 0.804  0.552  0.860  0.918 | **<0.001**  **<0.001**  **0.005**  **0.004** | 0.526  0.484  0.648  0.449 | 0.147  0.522  0.692  0.561 | 0.488  0.250  0.327  0.276 | 0.807  0.642  0.534  0.681 |

** Birthweight has been included in the explanatory models when 0.05 < p < 0.1 due to the significance of the variable in at least one data subset.*

*** Two models are given for the intention to treat group, as with one of number of days to transition to full suck feeds or CGA at full suck feeds is significant, but when both are included in an explanatory model, neither is significant.*
